# Effect of Vitamin D Supplementation on Inflammatory Markers in Non-Obese Lebanese Patients with Type 2 Diabetes: A Randomized Controlled Trial

**DOI:** 10.3390/nu12072033

**Published:** 2020-07-09

**Authors:** Cynthia El Hajj, Stéphane Walrand, Mariana Helou, Kaissar Yammine

**Affiliations:** 1 Department of Nutrition, Faculty of Natural Sciences, Lebanese American University, Beirut 1102 2801, Lebanon; 2 Unité de Nutrition Humaine, France Clermont Université, Université d’Auvergne, INRA, UMR 1019, UNH, CRNH Auvergne, 63009 Clermont-Ferrand, France; stephane.walrand@inrae.fr; 3 Department of Medicine, School of Medicine, Lebanese American University, Beirut 1102, Lebanon; marianahelou@hotmail.com; 4 Department of Orthopedic Surgery, Lebanese American University School of Medecine-Rizk Hospital, Beirut, Lebanon-Center for Evidence-Based Anatomy, Sports & Orthopedic Research, Jdeideh 1102, Lebanon; cesaryam@gmail.com

**Keywords:** type 2 diabetes mellitus, hs-CRP, IL-6, TNF-α, vitamin D

## Abstract

Background: A low serum 25-hydroxyvitamin D (25(OH) D) concentration has been associated with a higher risk of type 2 diabetes mellitus (T2DM), especially in older people. Our aim in this randomized controlled trial was to evaluate the effect of vitamin D treatment on inflammatory markers in non-obese Lebanese patients with T2DM, living in Beirut, Lebanon. Methods: Non-Obese patients with T2DM (*n* = 88), deficient/insufficient in vitamin D, were randomly assigned into one of two groups—a treatment group receiving 30,000 IU cholecalciferol/week for a period of six months, and a placebo group. Serum concentrations of TNF-α, high-sensitivity C-reactive protein (hs-CRP), and Interleukin-6 (IL-6) were the primary outcomes. A homeostatic model of insulin resistance (HOMA-IR) was assessed, in addition to serum concentrations of fasting blood glucose (FBG), HbA1C, (25(OH) D), and PTH. Results: The vitamin D group showed higher blood levels of (25(OH) D) (*p* < 0.0001), and a significant reduction in hs-CRP and TNF-α concentrations (*p* < 0.0001) compared to placebo. The decrease perceived in IL-6 concentrations was not significant (*p* = 0.1). No significant changes were seen in FBG (*p* = 0.9) and HbA1c levels (*p* = 0.85). Conclusion: Six months of vitamin D supplementation led to a decrease in some inflammatory markers in patients with T2DM. Additional studies with a larger sample and a longer period are advised in this regard. This trial was registered at ClinicalTrial.gov; Identifier number: NCT 03782805.

## 1. Introduction

Vitamin D deficiency was shown to be a key factor in the development of type 1, as well as type 2 diabetes [1]. Although Lebanon is among countries with abundant sunshine, vitamin D deficiency has been shown to be of significant prevalence among the population, regardless of their age group. According to Khalife et al. [2], the percentage of subjects deficient in vitamin D was found to be 57.86% in the area of greater Beirut, at a cut-off of 20 ng/mL and a mean of 19.89 ± 10.28 ng/mL. A previous study conducted on the elderly and adult Lebanese population also revealed similar results [3]. The International Diabetes Federation (IDF) has found that vitamin D deficiency and diabetes are both considered pandemics [4]. Recently, in 2019, the International Diabetes Federation Diabetes Atlas revealed that the global diabetes prevalence in 2019 was estimated to be 9.3% (463 million people), rising to 10.2% (578 million people) by 2030 and 10.9% (700 million) by 2045 [4]. Type 2 Diabetes Mellitus (T2DM) is a chronic metabolic disease that has become increasingly widespread. It is usually accompanied by a high blood sugar (hyperglycemia), which is considered one of the factors that stimulates inflammasome activation, and possibly a chronic inflammatory setting [5]. The prevalence of diabetes in Lebanon is an alarming health concern. The IDF estimated the prevalence of diabetes in Lebanon in 2015 at 122 per 1000 individuals [6]. In 2020, Bou Orm and Adib [7] revealed that the percentage of Lebanese diabetic adults is 7.95%. Besides, a large number of complications have been associated with type 2 diabetes, including disturbed cholesterol metabolism and dyslipidemia. The Lebanese results from the International Diabetes Management Practices Study Wave 6 presented that dyslipidemia was found to be prevalent among diabetic patients (68.4%), where 39.2% had a LDL <100 mg/dL and 56% had triglyceride levels <150 mg/dL. Genetic factors are involved in the development of T2DM, but many other environmental factors were shown to have the most significant contribution in the progress of this disease. These factors consist of a lack of physical activity, poor dietary habits, and obesity [8]. In this current study, we excluded obese patients with T2DM since well-documented connections were demonstrated between obesity and low vitamin D concentrations. Obese subjects are less exposed to sunlight due to lower exercise and physical activity [9]. Moreover, it is commonly acknowledged that adipose tissue is a tank for vitamin D in human subjects, and (25(OH) D) is sequestrated in adipose tissue [9]. Nevertheless, adipose tissue is recognized in producing adipokines, which could increase the risk of insulin resistance and, thus, lead to the development of T2DM [10,11,12].

Growing evidence has proposed that elevated blood concentrations of circulating inflammatory markers, such as interleukin-6 (IL-6), tumor necrosis factor-alpha (TNF-α), and high-sensitivity C-reactive protein (hs-CRP), can enhance the development of T2DM [13,14,15,16,17]. It has been revealed that vitamin D plays an important role in reducing inflammation and controlling immune activation [18,19,20]. Besides, vitamin D was shown to work on β-cells by preventing the production of pro-inflammatory cytokines and inhibiting their destruction, which lowers the prevalence of T2DM [21,22,23]. Furthermore, subjects deficient in vitamin D are shown to have higher levels of IL-6, TNF-α, and hs-CRP [24,25]. Vitamin D deficiency is now observed as a prospective risk factor for T2DM. A study conducted on Asian adults has found that the prevalence of type 2 diabetes is higher in subjects with vitamin D deficiency, compared to others with normal values of vitamin D [26]. Additionally, a new meta-analysis established that vitamin D administration may improve glycemic status in non-obese diabetic patients with vitamin D deficiency [18]. Another recent systematic review provided that vitamin D supplementation decreased HbA1c levels in diabetic adults [19]. We therefore assumed that vitamin D may be implicated in the mechanisms of systemic inflammation, which could assist in the progress of T2DM. Previous research has shown that adiponectin and adipokines (IL-6, TNF-α, and leptin) are related to a low vitamin D status and insulin resistance [20]. However, the mechanism involved in the reduction in inflammatory biomarkers in T2DM after vitamin D supplementation is not well established. Therefore, we conducted this randomized controlled trial (RCT) to observe the effect of cholecalciferol on inflammatory markers in non-obese Lebanese patients with type 2 Diabetes.

## 2. Subjects and Methods

### 2.1. Participants

Subjects meeting the criteria for the study were recruited from the endocrinology and family medicine outpatient clinics of Saint Charles Hospital (Fiyadiyeh, Lebanon) between May and July 2018. They were diabetic, non-obese (BMI = 18.5–29.8), and had vitamin D deficiency or insufficiency. According to IOM recommendations: vitamin D deficiency was defined as serum concentration (25(OH) D) < 12 ng/mL (30 nmoL/L); vitamin D insufficiency was defined as (25(OH) D), ranging from 12 to 20 ng/mL (50 nmoL/L). Criteria for exclusion were: obese subjects; subjects with hepatic disease, kidney disease, or hyperparathyroidism; subjects on vitamin D or calcium supplementation.

**Figure 1:**
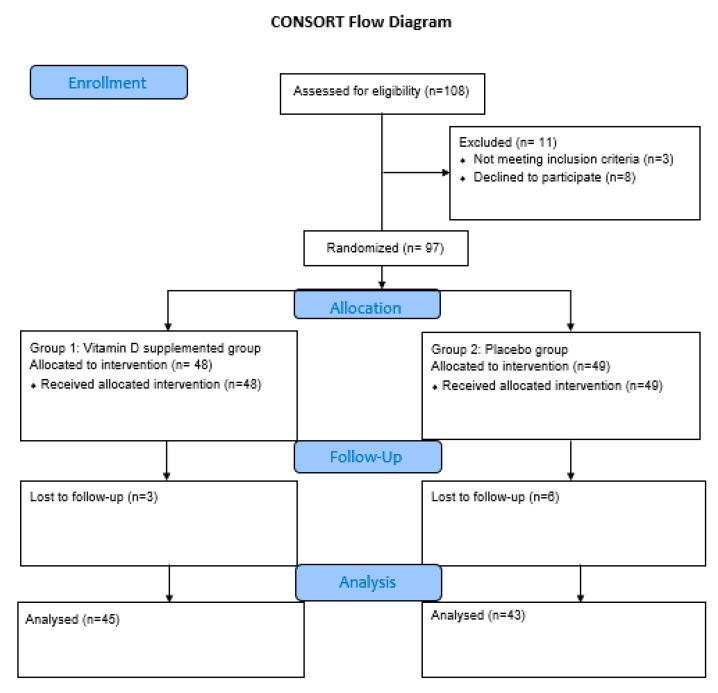
CONSORT flow diagram.

As shown in Figure 1, out of 108 recruited subjects, 11 were not included. The remaining 97 participants were randomly assigned into two groups—a treatment group receiving vitamin D supplements and a control group receiving a placebo pill. In total, 88 participants (45 men and 43 women) remained at the end of the study after those who lost at the follow-up (*n* = 9). The first visit was in July 2018. The subjects were asked about their exercise habits, the use of sun protection, and the duration of sun exposure during their daily life activities. Moreover, a short food frequency questionnaire was administered to assess their dietary vitamin D intake. Additionally, a written informed consent of the study, approved by the Ethics Committee of the institutional review board of Saint Charles hospital, was given to each subject. The protocols of this trial were prepared, based on the Declaration of Helsinki ethical principles for medical research involving human subjects.

### 2.2. Research Design and Supplementation Protocol

This trial was a randomized controlled double-blind study, where subjects were randomly allocated to take either a supplementation of 10,000 IU cholecalciferol (Euro-Pharm International, Saint-Léonard, QC, Canada) or a placebo pill (enclosing microcrystalline cellulose: 66.3%, starch: 33.2%, magnesium stearate: 0.5%, per serving) three times per week. According to Vieth et al. [27] and Bischoff-Ferrari et al. [28], 1000 IU (25 mcg) per day would benefit 50% of people and help them reach a vitamin D blood concentration of 33 ng/mL (82.4 nmoL/l). Taking 2000 IU (50 mcg) per day would be beneficial for almost everybody and help them reach a blood concentration of 33 ng/mL (82.4 nmoL/L). A dose of 4000 IU (100 mcg) of vitamin D per day would be the best for most deficient people and help them to reach healthy vitamin D blood concentrations. Moreover, the latest Consensus statement from the 2nd International Conference on Controversies in Vitamin D (2020) revealed that the upper limit (UL) for vitamin D in normal healthy individuals is 4000 IU/day (100 μg/day) [29]. Besides, high vitamin D dosage was given in many previous trials. In the ongoing Vitamin D and Type 2 Diabetes Study in the States, subjects were given a daily dose of 4000 IU to be taken for a period of three years [30]. Another recent trial studying the effects of vitamin D supplementation on cognitive function in people with type 2 diabetes gave the participants a very high dose of vitamin D_3_ (50,000 IU) to be taken every week over a three month period [31]. Based on these data, participants were asked to take the pills three times a week for a period of six months. A number from 1 to 88 was assigned to each participant, depending on his order of inclusion in the study. Randomization was performed using a simple randomization method by flipping a coin. This method was completed by a pharmacist and then subjects were allocated to their groups. The first group is the vitamin D group (*n* = 45), and the second is the placebo group (*n* = 43). Nobody (except for the pharmacist) was familiar with the group allocation. Besides, the pharmacist was in charge of the packing and coding of the supplements given to each subject. The first participant was assigned to the placebo group. The subjects were followed up by a phone call at three months of supplementation and were requested to come back for analysis after six months. Moreover, they were asked to give back the supplemental bottles (for counting) to check their compliance.

Laboratory assessments were completed at baseline and after six months of supplementation. As for the subjects’ medical histories and medications, they were evaluated using their doctors’ documents.

### 2.3. Laboratory Assessment

#### 2.3.1. Primary Outcomes

Subjects were asked to fast for 12 h before the blood tests. The serum samples were then frozen and warehoused at −80 °C in anticipation of the analysis. Sample analysis was performed at baseline and six months. Serum concentrations of TNF-α, high-sensitivity C-reactive protein (hs-CRP), and Interleukin-6 (IL-6) were the primary outcomes. For the inflammatory markers, serum concentrations of TNF-α were conducted using high-sensitivity ELISA kits (R&D Systems); inter-assay CV ∼8.4% and intra-essay CV ∼5.3%. Serum hs-CRP concentrations were completed through high-sensitivity ELISA CRP kits (Roche Diagnostics, UK); inter-assay CV ∼6.3% and intra-essay CV ∼7%. As for the serum concentrations of IL-6, they were measured using a Triturus ELISA analyzer (Grifols); inter-assay CV ∼7.7% and intra-assay CV ∼7.4%. The manufacture’s guidelines were respected while preparing the reagents.

#### 2.3.2. Secondary Outcomes

Fasting blood concentrations of vitamin D (25(OH) D), triglycerides (TG), total cholesterol (TC), HDL cholesterol (HDL-c), LDL cholesterol (LDL-c), fasting blood glucose (FBG), HbA1c, HOMA-IR, and PTH were the secondary outcomes. Serum concentrations of (25(OH) D) were completed using radioimmunoassay (DiaSorin, Stillwater, MN, USA); inter-assay CV <8% and intra-essay CV ∼4.9%. Our laboratory normal values for (25(OH) D) are 33–90 ng/mL. Hypovitaminosis is identified with a result <20 ng/mL (taking IOM references). TG, TC, HDL-c and LDL-c were measured enzymatically using a RA1000 autoanalyzer (Bayer Diagnostics, Suffolk, UK). FBG was accomplished through a hexokinase assay on blood transferred into specific tubes that are fluoridated; inter-assay CV 1.8% at 6.6 mmol/L. The hexokinase method, established by the American Association for Clinical Chemistry, is used most extensively in clinical research, and has been recognized as the reference method to assess blood glucose [32,33]. The values of HbA1c were evaluated using a Roche, Cobas 8000 (C701 and C702) analyzer. Insulin resistance (IR) was calculated by the homeostasis model assessment of insulin resistance using the following formula: HOMA-IR = [insulin (mU/L) × glucose (mg/dL)]/22.5. PTH was assessed by immunoradiometric assay (a two side one) including a NH_2_-terminal antibody meant for conservation.

### 2.4. Anthropometry Assessment

Body mass index (BMI) and waist circumference (WC) were evaluated at baseline and after six months of cholecalciferol administration. BMI calculation was accomplished using the recognized formula: body weight (kg) divided by the square of body height (m²) [kg/m²]. Weight and height were measured via the body composition analyzer (Tanita BC-418 Segmental Body Composition Analyzer, Arlington Heights, IL, USA). Subjects with BMI ≤ 25 kg/m^2^ were categorized as having an ideal or normal weight. Waist circumference was assessed using a measuring tape in centimeters (cm) while the subject was standing. The value was taken straight at the level of the belly button. Blood pressure was measured using the NPB-3900, Nellcor automated device (Puritan Bennett, Pleasanton, CA, USA). Subjects were asked to be seated for a minimum of 5 min and the measurement was done using the bicep (upper arm) of the right arm.

### 2.5. Statistical Analysis

Statistical calculations were computed using the Statistical Package for Social Sciences (SPSS Inc., Chicago, IL, USA, version 21). The baseline characteristics of the participants are stated as means ± SD for all the parameters and as a percentage for the HbA1c variable. The primary outcomes of the study were the changes observed in serum concentrations of TNF-α, hs-CRP, and IL-6 from baseline and after six months of administration of vitamin D. Data distribution was checked for normality; histograms and the Shapiro–Wilk Normality Test demonstrated a normal distribution.

The evaluation of the mean changes within the same group (from baseline and after the sixth month) was completed using the paired-samples *t*-test, while two-way ANOVA analysis was used for group comparison. Multiple testing was conducted for each outcome comparison. Multiple linear regression was completed to establish the relationship between (25(OH) D) with all the parameters of the present study. A *p*-value of <0.05 was considered statistically significant.

## 3. Results

### 3.1. Anthropometric Characteristics of the Study’s Subjects

In total, 88 participants were randomly allocated to one of the two groups—the treatment group (*n* = 45) or the placebo group (*n* = 43). The study population included 45 men and 43 women with T2DM. The mean age of the total sample was of 66.3 (±4.4) years, and the mean BMI was 22.9 (±1.93). The anthropometric characteristics of both groups at baseline were similar, and their comparison (Vitamin D group vs. placebo group) was not significant.

### 3.2. Study Outcomes

Table 1 and Table 2 show the mean change data before and after intervention. Serum concentration of (25(OH) D) at six months increased only in the vitamin D group; the (25(OH) D) level was significantly higher (by 20.1 ng/mL) after supplementation compared to the placebo (*p* < 0.0001). BMI and waist circumference showed a significant decrease (*p* = 0.0449 and *p* = 0.001, respectively) at six months in the treatment group. Body fat (%) also showed a significant decrease (*p* = 0.0001). Systolic and diastolic blood pressure did not present any significant change (*p* = 0.34 and 0.21, respectively). Similarly, no significant change was noted for serum concentrations of FBG (*p* = 0.09), HbA1c (*p* = 0.85), and HOMA-IR (*p* = 0.96). PTH concentrations showed significant changes after vitamin D treatment (*p* < 0.0001). Serum TG levels presented a significant decrease in the treatment group (*p* = 0.02) compared to placebo. Additionally, HDL-c levels were significantly increased after vitamin D supplementation (*p* = 0.022). The changes observed in serum TC and LDL-c were not significant (*p* = 0.38 and 0.18, respectively). As for the inflammatory markers, six months of vitamin D supplementation led to a significant decrease in CRP level, found in the vitamin D group (5.35 ± 2.4 to 2.84 ± 1.85 (*p* < 0.0001)) compared to the placebo group (5.25 ± 2.47 to 5 ± 2.7 (*p* = 0.6)). Likewise, a significant decrease in TNF-α level was found in the vitamin D group (9.82 ± 1.17 to 5.15 ± 1.12 (*p* < 0.0001)) compared to the placebo group (10.14 ± 1.82 to 9.32 ± 1.21 (*p* = 0.15)). However, the change in IL-6 concentration was not significant.

#### 3.2.1. Predictors of Inflammatory Marker Changes at Six Months

In univariate analysis, only baseline values and vitamin D at six months were correlated with all three inflammation markers at six months. In multivariate analysis, including all other variables, only baseline values of the inflammation markers were correlated with their values at six months. No gender difference was seen after supplementation.

#### 3.2.2. Post Hoc Analyses

When running univariate and multivariate analyses, no relationship was shown between the baseline mean values of inflammatory markers and age, gender, or BMI for all the subjects, as well as for each group of the study.

After six months with vitamin D treatment, a beneficial effect was perceived on two inflammatory markers (TNF-α and CRP), but not on IL-6 levels. Though multivariate analyses showed no significance in relation to vitamin D values at six months, all corresponding *p*-values were between 0.68 and 0.31, far less than the *p*- of all other variables. Interestingly, after running the ANCOVA model, including vitamin D deficient and insufficient subjects, the effect of vitamin D treatment was higher in the deficient vs. insufficient group. The mean change in 25(OH) D was higher in the deficient (20.8 ± 3.6) vs. insufficient (17.6 ± 3.2) group. This was similar for TNF-α (−0.48 ± 1.01 vs. −0.38 ± 1.02) and hs-CRP (−2.83 ± 2.16 vs. −2.13 ± 2.51). Estimated mean change for each group from the ANCOVA model is shown in Table 3.

## 4. Discussion

The aim of this randomized controlled trial was to study the relationship between vitamin D status and inflammatory markers in non-obese Lebanese patients with T2DM. Our findings revealed that improving vitamin D status by supplementation resulted in the amelioration of some inflammatory markers (TNF-α and CRP) in patients with T2DM. To our knowledge, this is the first Lebanese study to examine the relationship between vitamin D status and inflammatory markers in non-obese adults with T2DM. Besides, this study is the first to evaluate the effect of vitamin D treatment in a Lebanese population, which was shown to be a high-risk population, since vitamin D deficiency is highly prevalent among its residents and the incidence of T2DM is shown to be important (14.6% in 2017 [34]). It is true that our experimental methods were based on previous studies, but a randomized controlled trial studying the effect of vitamin D treatment on inflammatory markers among the Lebanese population, in particular among diabetic patients, was never considered before.

Our results showed no significant effect of vitamin D administration on HbA1c, even though a positive association was revealed between (25(OH) D) status and BMI. These results came in accordance with our previous study [35], that showed no relationship between (25(OH) D) status and HbA1c. Of note, our subjects had a lower BMI, while waist circumference was relatively high. According to Tang et al. [36], even persons with normal BMI but with central abdominal obesity might place them at risk for non-obese diabetes. In fact, abdominal obesity has been proven to be the best predictor of type 2 diabetes, compared to BMI, waist/hip ratio, and other anthropometric measurements. The development of type 2 diabetes could be also related to hypertriglyceridemia and low HDL-cholesterol levels. Our subjects had a high triglycerides concentrations and low HDL-cholesterol levels, which were ameliorated after vitamin D treatment. These specific clinical changes show that vitamin D supplementation may be useful in hypertriglyceridemia patients with vitamin D deficiency, who are at high risk of cardiovascular diseases. Moreover, improvements in HDL cholesterol levels can similarly help reduce the risk of heart disease, especially in diabetic patients who are at higher risk of developing heart disease, compared to healthy adults [37].

In the present study, the subjects were deficient in vitamin D for many reasons. They had barely any direct sun exposure and supplementation was not advised to them. In addition, the average vitamin D intake according to our food frequency questionnaire [38] was 1.28 ± 0.14 µg/d.

As T2DM starts to develop, the body comes to be less sensitive to insulin thus resulting in insulin resistance, which contributes to inflammation. Nikooyeh et al. [39] concluded that inflammatory processes taking place in adipocytes may lead to systemic low-grade inflammation, causing insulin resistance and diabetes.

Interestingly, in our study, an improvement in (25(OH) D) status resulted in a significant decrease in serum TNF-α concentrations. In support of our results, the study of Ghavamzadeh et al. [40] has shown that vitamin D3 (400 IU/d) for 14 weeks resulted in a significant decrease in TNF-α concentrations. Another study, conducted by Inanir et al. [41], revealed similar results; a decrease in TNF-α concentrations after calcitriol supplementation for six months in postmenopausal women with osteoporosis. These results also agree with other experimental studies [24,42], proving that vitamin D supplementation helps in reducing inflammatory cytokines concentrations, such as TNF-α, and consequently decrease the risk of insulin resistance development This supports the concept that vitamin D can enhance insulin secretion by improving calcium flux in pancreatic β cells.

CRP is considered as the most frequent marker that measures low-grade inflammation. Recent studies in subjects with type 2 diabetes came in accordance with our results and revealed the beneficial effect on serum hs-CRP after vitamin D supplementation. One research study has shown that diabetic patients taking vitamin D supplementation for a period of six months decreased serum hs-CRP by −1.0 ± 0.5, compared with placebo +0.6 ± 0.5 μg/mL (*p* = 0.02) [42]. A recent systematic review and a meta-analysis of randomized controlled trials, published in 2018, concluded that vitamin D supplementation decreases serum hs-CRP in T2DM subjects [43]. One mechanism that could explain the effect of vitamin D supplementation on CRP reduction is that Vitamin D_3_ activates the expression of the inhibitory protein NF-κB (nuclear factor kappa B) and subsequently contributes to a reduction in serum hs-CRP [44]. In addition, when vitamin D binds to its receptors in monocytes, the production of pro-inflammatory cytokines decreases. Thus, vitamin D helps in reducing the concentration of CRP and other inflammatory markers [45].

Furthermore, previous studies disclosed that vitamin D might prevent the production of IL-6 [45,46,47] and thus could assist in reducing the incidence of type 2 diabetes mellitus [48]. Stepanova et al. [49] concluded in their recent single-center randomized trial that the group receiving 40,000 IU cholecalciferol per week for a period of six months showed an improvement in serum IL-6 (*p* = 0.017). Unfortunately, within this analysis, vitamin D treatment did not support this hypothesis, since the changes observed in IL-6 concentrations were not significant in our T2DM subjects. This could be related to the fact that the dosage of vitamin D was lower in our study (30,000 IU/week). Nevertheless, our results were analogous with the outcomes of Yu et al. [43], which presented no significant influence of vitamin D supplementation on IL-6 concentrations in T2DM subjects.

Our results show that six-month vitamin D supplementation had a beneficial effect on blood triglycerides levels and HDL-c. These findings were supported by previous trials that presented an amelioration in serum triglyceride levels and HDL-c after vitamin D treatment [50,51,52,53]. Several mechanisms have been acknowledged that may somehow clarify the effects of vitamin D on blood lipids. In vitro, lipoprotein lipase (an enzyme that hydrolyzes triglycerides in lipoproteins) is regulated by vitamin D metabolites [51], which help by increasing HDL-c and lowering triglyceride concentrations. Additionally, vitamin D was shown to reduce insulin resistance because of its anti-inflammatory effects and might, consequently, reduce low-grade chronic inflammation [52,53], which may lower triglyceride levels and increase HDL-c. Additionally, a low vitamin D level increases PTH level, which boosts lipogenesis and reduces lipolysis, causing a high TG level. On the contrary, when the vitamin D level is high, PTH decreases, leading to an increase in lipolysis and, thus, a decrease in TG level [53].

However, we found no significant effect of vitamin D treatment on blood pressure, TC, and LDL-c, irrespective of baseline serum 25(OH) D concentrations. Although these results came in contrast with some other published studies, they are in agreement with the recently published study by Kubiak et al. [54]. Similarly, another randomized control trial, conducted by Seibert et al. [55], showed no effect of vitamin D supplementation on blood pressure, TC, and LDL-c.

The main strength of this work is that it is a randomized, double-blind, placebo-controlled trial. It is the first study to assess the effect of vitamin D treatment on inflammatory markers in non-obese Lebanese patients with T2DM. Luckily, participants who were lost at follow-up were minimal; about 92% of the allocated subjects completed the study, which lessened the probability of related bias. Nevertheless, we had a main limitation, which was the duration of supplementation. It was short-term, though it was suitable for recognizing positive improvements in some inflammatory markers.

## 5. Conclusions

In conclusion, the present study supports the hypothesis that vitamin D administration at a dose of 30,000 IU per week improves vitamin D status, and some inflammatory markers (tumor necrosis factor alpha and C-reactive protein) in patients with T2DM. However, our findings show no significant relationship between vitamin D status and serum IL-6 concentrations. That is why further studies with larger sample sizes are needed to confirm the anti-inflammatory effect of vitamin D. Besides, additional research will help in expanding our understanding of how vitamin D improves inflammation in patients with T2DM.

## Figures and Tables

**Table 1:** Changes in primary outcomes of the participants after 6 months of vitamin D supplementation.

	Vitamin D Group (*n* = 45)				Placebo Group (*n* = 43)				Between Group Difference
Variable	Baseline	After Supplementation	Mean Change (95% CI)	* *p* Value	Before	After Supplementation	Mean Change (95% CI)	* *p* Value	^†^*p* Value
Sample size	45	-	-	-	43	-	-	-	-
Gender (M/F)	23M/22F	-	-	-	22M/21F	-	-	-	-
Age (years)	66.9 ± 4.1	-	-	-	65.7 ± 4.5	-	-	-	0.2
Diabetes Duration (years)	8.7	-	-	-	8.5	-	-	-	-
TNF-α (pg/mL)	3.05 ± 1.02	2.61 ± 1.04	−0.44	0.0001	2.97 ± 0.98	3.01 ± 0.83	0.04	0.68	<0.0001
hs-CRP (ng/mL)	5.25 ± 2.47	2.84 ± 2.14	−2.41	0.0001	5.3 ± 2.31	5.19 ± 2.23	−0.12	0.34	<0.0001
IL-6 (pg/mL)	3.86 ± 1.7	3.15 ± 1.8	−0.71	0.27	4.11 ± 1.8	4.21 ± 1.6	−0.1	0.31	0.94

* Paired-Samples *t*-test was performed to evaluate the changes (from baseline to six months) in the primary outcomes within the same group; **^†^** two-way ANOVA analysis was performed for between group comparisons; significance when *p ≤* 0.05. TNF-α, tumor necrosis factor-alpha; hs-CRP, high-sensitivity C-reactive protein; IL-6, interleukin-6.

**Table 2:** Changes in secondary outcomes of the participants after six months of vitamin D supplementation.

	Vitamin D Group (*n* = 45)				Placebo Group (*n* = 43)				Between Group Difference
Variable	Baseline	After Supplementation	Mean Change (95% CI)	* *p* Value	Baseline	After Supplementation	Mean Change (95% CI)	* *p* Value	^†^*p* Value
25(OH)D (ng/mL)	14.8 ± 4.5	34.9 ± 4.7	20.1	<0.0001	15.02 ± 4.2	14.5 ± 3.9	−0.52	0.96	<0.0001
BMI (kg/m^2^)	22.6 ± 1.72	21.2 ± 1.1	−1.4	<0.0001	23.2 ± 5.71	24.1 ± 4.89	0.9	0.08	<0.0001
Waist circumference (cm)	91.5 ± 5.88	88.3 ± 5.63	−3.2	0.0001	92.2 ± 5.62	93.9 ± 5.14	0.4	0.21	0.0001
Body fat (%)	29.3 ± 6.40	27.9 ± 6.50	−1.4	0.0001	30.41 ± 5.33	30.92 ± 4.36	0.51	0.13	0.05
Systolic BP (mmHg)	141 ± 2.8	140 ± 2.2	0.9	0.58	144 ± 2.5	143 ± 2.3	−1	0.18	0.34
Diastolic BP (mmHg)	84 ± 2.1	86 ± 2.2	1.7	0.31	86 ± 1.9	85 ± 1.7	−0.9	0.24	0.21
TG (mg/dL)	242.8 ± 9.9	211.2 ± 8.8	−31.2	0.0001	244.2 ± 8.78	240.2 ± 8.18	−3.9	0.46	0.02
TC (mg/dL)	180.2 ± 0.99	179.4 ± 1.2	−0.6	0.21	182.62 ± 1.1	182.1 ± 1.2	−0.02	0.12	0.38
HDL-c (mg/dL)	32.31 ± 1.26	35.4 ± 1.12	3.02	0.0001	31.81 ± 1.15	31.42 ± 1.08	−0.49	0.11	0.022
LDL-c (mg/dL)	145.5 ± 4.27	140.9 ± 1.2	−4.4	0.041	146.3 ± 5.01	145.9 ± 5.2	−0.8	0.32	0.18
FBG (mg/dL)	184.6 ± 0.8	184.5 ± 0.9	−0.1	0.11	185.14 ± 0.8	185.09 ± 0.7	0.05	0.95	0.84
HbA1c (%)	6.59 ± 0.64	6.53 ± 0.63	−0.06	0.38	6.82 ± 1.27	6.64 ± 1.31	−0.18	0.31	0.31
HOMA-IR	2.85 ± 2.55	2.51 ± 2.46	−0.34	0.27	2.66 ± 1.91	2.41 ± 1.92	−0.25	0.96	0.26
PTH (ng/L)	37.5 ± 3.8	30.7 ± 3.7	−6.7	<0.0001	36.25 ± 8.04	36.1 ± 7.56	−0.15	0.17	<0.0001

* Paired-Samples *t*-test was performed to evaluate the changes (from baseline to 6 months) in the secondary outcomes within the same group; ^†^ two-way ANOVA analysis was performed for between group comparisons; significance when *p* ≤ 0.05. BMI; body mass index, BP; blood pressure, TG; triglycerides, TC; total cholesterol, HDL-c; HDL cholesterol, LDL-c; LDL cholesterol, FBG; fasting blood glucose, HbA1c; hemoglobin A1c, HOMA-IR; homeostatic model assessment of insulin resistance; PTH: parathyroid hormone.

**Table 3:** Effect of vitamin D supplementation on mean change in TNF-α, hs-CRP, IL-6, and 25(OH) D in deficient vs. insufficient subjects using (ANCOVA).

Mean Change		Vitamin D Deficient (*n* = 16) Insufficient (*n* = 29)	Placebo Deficient (*n* = 15) Insufficient (*n* = 28)	*p*-Value
TNF-α	Deficient	−0.48 ± 1.01	0.03 ± 0.78	<0.001
	Insufficient	−0.38 ±1.02	0.05 ± 0.8	<0.001
hs-CRP	Deficient	−2.83 ± 2.16	−0.12 ± 2.21	<0.001
	Insufficient	−2.13 ± 2.51	−0.11 ± 2.30	<0.001
IL-6	Deficient	−0.73 ± 1.8	−0.18 ± 1.6	0.92
	Insufficient	−0.66 ± 1.7	−0.09 ± 1.7	0.96
25(OH)D	Deficient	20.8 ± 3.6	−0.01 ± 3.6	<0.001
	Insufficient	17.6 ± 3.2	−0.03 ± 3.8	<0.001

Data are expressed as mean ± SD. *p*-values are for differences in mean change between Vitamin D and Placebo groups. TNF-α, tumor necrosis factor-alpha; hs-CRP, high-sensitivity C-reactive protein; IL-6, interleukin-6.

## Abbreviations

25(OH) D, 25-hydroxyvitamin D; BMI, body mass index; BP, blood pressure; FBG, fasting blood glucose; HbA1c, glycated hemoglobin; HOMA, homeostatic model assessment; hs-CRP, high sensitive C-reactive protein; IL-6, interleukin 6; IR, insulin resistance; PTH, parathyroid hormone; TC, total cholesterol; HDL-c, HDL cholesterol; LDL-c, LDL cholesterol; TG, triglycerides; TNF-α, tumor necrosis factor alpha.

## References

[1] Bland R., Markovic D., Hill C.E., Hughes S. (2004). Expression of 25-hydroxyvitamin D3-1 alpha-hydroxylase in pancreatic islets. J. Steroid Biochem. Mol. Biol..

[2] Khalife H., Khalife H., Omyri H., Khalife H., Abdel-Sater F. (2017). Vitamin D deficiency among the healthy population in Lebanon. World J. Pharm. Pharm. Sci..

[3] El-Rassi R., Baliki G., Fulheihan G.E.H. (2009). Vitanim D Status in Middle East and Africa.

[4] (2019). International Diabetes Federation: IDF Diabetes Atlas. https://www.diabetesatlas.org/upload/resources/2019/IDFAtlas9th_Edition_2019.pdf.

[5] Sepehri Z., Kiani Z., Afshari M., Kohan F., Dalvand A., Ghavami S. (2017). Inflammasomes and type 2 diabetes: An updated systemic review. Immunol. Lett..

[6] International Diabetes Federation (2015). Diabetes Atlas 2015.

[7] Bou-Orm I., Adib S. (2020). Prevalence and clinical characteristics of diabetes mellitus in Lebanon: A national survey. East Mediterr. Health J..

[8] Zhao L.M., Tian X.Q., Ge J.P., Xu Y.C. (2013). Vitamin D intake and type 2 diabetes risk: A meta-analysis of prospective cohort studies. Afr. Health Sci..

[9] Blum M., Dolnikowski G., Seyoum E., Harris S.S., Booth S.L., Peterson J., Dawson-Hughes B. (2008). Vitamin D3 in fat tissue. Endocrine.

[10] Alberti K.G.M.M., Eckel R.H., Grundy S.M., Zimmet P.Z., Cleeman J.I., Donato K.A., Fruchart J.-C., James P.T., Loria C.M., Smith S.C. (2009). Harmonizing the metabolic syndrome: A joint interim statement of the International Diabetes Federation Task Force on Epidemiology and Prevention; National Heart, Lung, and Blood Institute; American Heart Association for the Study of Obesity. Circulation.

[11] Garcia C., Feve B., Ferre P., Halimi S., Baizri H., Bordier L., Guiu G., Dupuy O., Bauduceau B., Mayaudon H. (2010). Diabetes and inflammation: Fundamental aspects and clinical implications. Diabetes Metab..

[12] McGill T., Stewart J.M., Lithander F., Strik C.M., Poppitt S.D. (2008). Relationship of low serum vitamin D_3_ with anthropometry and markers of the metabolic syndrome and diabetes in overweight and obesity. Nutr. J..

[13] Velloso L.A., Eizirik D.L., Cnop M. (2013). Type 2 diabetes mellitus-an autoimmune disease?. Nat. Rev. Endocrinol..

[14] Donath M.Y., Shoelson S.E. (2011). Type 2 diabetes as an inflammatory disease. Nat. Rev. Immunol..

[15] Laird E., McNulty H., Ward M., Hoey L., McSorley E., Wallace J.M.W., Caeson E., Molly A.M., Healy M., Cunningham C. (2014). Vitamin D deficiency is associated with inflammation in older Irish adults. J. Clin. Endocrinol. Metab..

[16] Akash M.S., Rehman K., Chen S. (2013). Role of inflammatory mechanisms in pathogenesis of type 2 diabetes mellitus. J. Cell. Biochem..

[17] Dutta D., Mondal S.A., Choudhuri S., Maisnam I., Reza A.H.H., Bhattacharya B., Chowdhury S., Mukhopadhyay S. (2014). Vitamin-D supplementation in prediabetes reduced progression to type 2 diabetes and was associated with decreased insulin resistance and systemic inflammation: An open label randomized prospective study from Eastern India. Diabetes Res. Clin. Pract..

[18] Wu C., Qiu S., Zhu X., Li L. (2017). Vitamin D supplementation and glycemic control in type 2 diabetes patients: A systematic review and meta-analysis. Metabolism.

[19] Lee C.J., Iyer G., Liu Y., Kalyani R.R., Ligon C.B., Varma S., Mathioudakis N. (2017). The effect of vitamin D supplementation on glucose metabolism in type 2 diabetes mellitus: A systematic review and meta-analysis of intervention studies. J. Diabetes Complicat..

[20] Nimitphong H., Chanprasertyothin S., Jongjaroenprasert W., Ongphiphadhanakul B. (2009). The association between vitamin D status and circulating adiponectin independent of adiposity in subjects with abnormal glucose tolerance. Endocrine.

[21] Alvarez J.A., Ashraf A. (2010). Role of vitamin D in insulin secretion and insulin sensitivity for glucose homeostasis. Int. J. Endocrinol..

[22] Husemoen L.L.N., Skaaby T., Thuesen B.H., Jørgensen T., Fenger R.V., Linneberg A. (2012). Serum (25(OH) D) and incident type 2 diabetes: A cohort study. Eur. J. Clin. Nutr..

[23] Forouhi N.G., Ye Z., Rickard A.P., Khaw K.T., Luben R., Langenberg C., Wareham N.J. (2012). Circulating 25-hydroxyvitamin D concentration and the risk of type 2 diabetes: Results from the European Prospective Investigation into Cancer (EPIC)-Norfolk cohort and updates meta-analysis of prospective studies. Diabetologia.

[24] Peterson C.A., Heffernan M.E. (2008). Serum tumor necrosis factor-alpha concentrations are negatively correlated with serum (25(OH) D) concentrations in healthy women. J. Inflamm..

[25] De Vita F., Lauretani F., Bauer J., Bautmans I., Shardell M., Cherubini A., Bondi G., Zuliani G., Bandineli S., Dall’Aglio E. (2014). Relationship between vitamin D and inflammatory markers in older individuals. Age.

[26] Lim S., Kim M.J., Choi S.H., Shin C.S., Park K.S., Jang H.C., Billings L.K., Meigs J.B. (2013). Association of vitamin D deficiency with incidence of type 2 diabetes in high-risk Asian subjects. Am. J. Clin. Nutr..

[27] Vieth R., Bischoff-Ferrari H., Boucher B.J. (2007). The urgent need to recommend an intake of vitamin D that is effective. Am. J. Clin. Nutr..

[28] Bischoff-Ferrari H.A., Giovannucci E., Willett W.C., Dietrich T., Dawson-Hughes B. (2006). Estimation of optimal serum concentrations of 25-hydroxyvitamin D for multiple health outcomes. Am. J. Clin. Nutr..

[29] Giustina A., Adler R.A., Binkley N., Bollerslev J., Bouillon R., Dawson-Hughes B., Ebeling P.R., Feldman D., Formenti A.M., Marcocci C. (2020). Consensus statement from 2nd International Conference on Controversies in Vitamin D. Rev. Endocr. Metab. Disord..

[30] Pittas A.G., Dawson-Hughes B., Sheehan P.R., Rosen C.J., Ware J.H., Knowler W.C., D2d Research Group (2014). Rationale and Design of the Vitamin D and Type 2 Diabetes (D2d) Study: A Diabetes Prevention Trial. Diabetes Care.

[31] Byrn M.A., Adams W., Penckofer S., Emanuele M.A. (2019). Vitamin D Supplementation and Cognition in People with Type 2 Diabetes: A Randomized Control Trial. J. Diabetes Res..

[32] Hagvik J. (2007). Glucose Measurement: Time for a Gold Standard. J. Diabetes Sci. Technol..

[33] Ambade V.M., Sharma Y.V., Somani B.L. (1998). Methods for estimation of blood glucose a comparative evaluation. Med. J. Armed. Forces India.

[34] Ahmadieh H., Itani H., Itani S., Sidani K., Kassem M., Farhat K., Jbeily M., Itani A. (2018). Diabetes and depression in Lebanon and association with glycemic control: A cross-sectional study. Dovepress.

[35] El Hajj C., Chardigny J.M., Boirie Y., Yammine K., Helou M., Walrand S. (2018). Effect of vitamin D treatment on glucose homeostasis and metabolism in Lebanese older adults: A randomized controlled trial. J. Nutr. Health Aging.

[36] Tang Z., Fang Z., Huang W., Liu Z., Chen Y., Li Z., Zhu T., Wang Q., Simpson S., Lin R. (2016). Non-Obese Diabetes and Its Associated Factors in an Underdeveloped Area of South China, Guangxi. Int. J. Environ. Res. Public Health.

[37] National Centers for Disease Control and Prevention (2014). National Diabetes Statistics Report. http://www.cdc.gov/diabetes/data/statistics/statistics-report.html.

[38] Hedlund L., Brekke H.K., Brembeck P., Augustin H. (2014). A Short Questionnaire for Assessment of Dietary Vitamin D Intake. Eur. J. Nutr. Food Saf..

[39] Nikooyeh B., Neyestani T.R., Tayebinejad N., Alavi-Majd H., Shariatzadeh N., Kalayi A., Zahedirad M., Heravifard S., Salekzamani S. (2014). Daily intake of vitamin D- or calcium vitamin D-fortified Persian yogurt drink (doogh) attenuates diabetes-induced oxidative stress: Evidence for antioxidative properties of vitamin D. J. Hum. Nutr. Diet.

[40] Ghavamzadeh S., Mobasseri M., Mahdavi R. (2014). The Effect of Vitamin D Supplementation on Adiposity, Blood Glycated Hemoglobin, Serum Leptin and Tumor Necrosis Factor-α in Type 2 Diabetic Patients. Int. J. Prev. Med..

[41] Inanir A., Ozoran K., Tutkak H., Mermerci B. (2004). The effects of calcitriol therapy on serum interleukin-1, interleukin-6 and tumour necrosis factor-alpha concentrations in post-menopausal patients with osteoporosis. J. Int. Med. Res..

[42] Farrokhian A., Raygan F., Bahmani F., Talari H.R., Esfandiari R., Esmaillzadeh A., Asemi Z. (2017). Long-Term Vitamin D Supplementation Affects Metabolic Status in Vitamin D–Deficient Type 2 Diabetic Patients with Coronary Artery Disease. J. Nutr..

[43] Yu Y., Tian L., Xiao Y., Huang G., Zhang M. (2018). Effect of Vitamin D Supplementation on Some Inflammatory Biomarkers in Type 2 Diabetes Mellitus Subjects: A Systematic Review and Meta-Analysis of Randomized Controlled Trials. Ann. Nutr. Metab..

[44] Ngo D.T., Sverdlov A.L., McNeil J.J., Horowitz J.D. (2010). Does vitamin D modulate asymmetric dimethylarginine and C-reactive protein concentrations?. Am. J. Med..

[45] Colin E.M., Asmawidjaja P.S., van Hamburg J.P., Mus A.M., van Driel M., Hazes J.M., van Leeuwen J.P., Lubberts E. (2010). 1, 25-Dihydroxyvitamin D3 modulates Th17 polarization and interleukin-22 expression by memory T cells from patients with early rheumatoid arthritis. Arth. Rheum..

[46] Khoo A.L., Chai L.Y., Koenen H.J., Sweep F.C., Joosten I., Netea M.G., van der Ven A.J. (2011). Regulation of cytokine responses by seasonality of vitamin D status in healthy individuals. Clin. Exp. Immunol..

[47] Müller K., Diamant M., Bendtzen K. (1991). Inhibition of production and function of interleukin-6 by 1, 25-dihydroxyvitamin D3. Immunol. Lett..

[48] Campbell I.L., Kay T.W., Oxbrow L., Harrison L.C. (1991). Essential role for interferon-gamma and interleukin-6 in autoimmune insulin-dependent diabetes in NOD/Wehi mice. J. Clin. Investig..

[49] Stepanova A.P., Karonova T.L., Galagoudza M., Vasileva E.Y., Jude E.B. (2019). The Effect of Vitamin D Supplementation on the Cytokines Levels in Patients with Type 2 Diabetes Mellitus and Diabetic Neuropathy. Diabetes.

[50] Querfeld U., Hoffmann M., Klaus G., Eifinger F., Ackerschott M., Michalk D., Kern P.A. (1999). Antagonistic effects of vitamin D and parathyroid hormone on lipoprotein lipase in cultured adipocytes. J. Am. Soc. Nephrol..

[51] Hewison M. (2012). An update on vitamin D and human immunity. Clin. Endocrinol..

[52] Chagas C.E., Borges M.C., Martini L.A., Rogero M.M. (2012). Focus on vitamin D, inflammation and type 2 diabetes. Nutrients.

[53] Zittermann A., Frisch S., Berthold H.K., Gotting C., Kuhn J., Kleesiek K., Stehle P., Koertke H., Koerfer R. (2009). Vitamin D supplementation enhances the beneficial effects of weight loss on cardiovascular disease risk markers. Am. J. Clin. Nutr..

[54] Kubiak J., Thorsby P.M., Kamycheva E., Jorde R. (2018). Vitamin D supplementation does not improve CVD risk factors in vitamin D-insufficient subjects. Endocr. Connect..

[55] Seibert E., Lehmann U., Riedel A., Ulrich C., Hirche F., Brandsch C., Stangl G.I. (2017). Vitamin D_3_ supplementation does not modify cardiovascular risk profile of adults with inadequate vitamin D status. Eur. J. Nutr..

